# Machine Learning in Primary Health Care: The Research Landscape

**DOI:** 10.3390/healthcare13131629

**Published:** 2025-07-07

**Authors:** Jernej Završnik, Peter Kokol, Bojan Žlahtič, Helena Blažun Vošner

**Affiliations:** 1 Community Healthcare Center Dr. Adolf Drolc Maribor, 2000 Maribor, Slovenia; jernej.zavrsnik@zd-mb.si (J.Z.); helena.blazun@zd-mb.si (H.B.V.); 2 Alma Mater Europaea, 2000 Maribor, Slovenia; 3 Faculty of Electrical Engineering and Computer Science, University of Maribor, 2000 Maribor, Slovenia

**Keywords:** primary health care, machine learning, research landscape, synthetic knowledge synthesis

## Abstract

**Background:** Artificial intelligence and machine learning are playing crucial roles in digital transformation, aiming to improve the efficiency, effectiveness, equity, and responsiveness of primary health systems and their services. **Method:** Using synthetic knowledge synthesis and bibliometric and thematic analysis triangulation, we identified the most productive and prolific countries, institutions, funding sponsors, source titles, publications productivity trends, and principal research categories and themes. **Results:** The United States and the United Kingdom were the most productive countries; *Plos One* and *BJM Open* were the most prolific journals; and the National Institutes of Health, USA, and the National Natural Science Foundation of China were the most productive funding sponsors. The publication productivity trend is positive and exponential. The main themes are related to natural language processing in clinical decision-making, primary health care optimization focusing on early diagnosis and screening, improving health-based social determinants, and using chatbots to optimize communications with patients and between health professionals. **Conclusions:** The use of machine learning in primary health care aims to address the significant global burden of so-called “missed diagnostic opportunities” while minimizing possible adverse effects on patients.

## 1. Introduction

The development of digital health can empower equitable access to global expert-level health care and transform health care into a more value-based, equitable, and patient-centric system [1,2]. Artificial intelligence is essential to this transformation at both the general [3] and primary health care levels [4,5]. The use of machine learning, an essential part of artificial intelligence, is already showing promising results in primary health care [6,7,8,9,10,11,12]. In general, the use of machine learning in health care can improve efficiency by improving the following factors: health care service delivery [13,14], screening [15,16], health care cost management [17], equity by predicting missing appointments [13,18] or improving access to primary health care [19,20], responsiveness through better decision-making [21,22], and the monitoring of primary health services [23]. Recently, several reviews on machine learning use in primary health care have been published. However, these reviews were not oriented toward primary health as a whole but were limited to specific diseases, conditions, prognostic or prediction modeling, or specific health care services [21,24,25,26,27,28]. Consequently, due to the multidisciplinary and multilayered nature of the use of machine learning in primary health care, a holistic and systemic research landscape of this research area is needed

- To identify the most prolific machine learning methods and the primary health care research categories and themes where these methods are applied;
- To identify publishing venues where researchers can be informed about the use of AI in primary health care and where they can publish the outcomes of their research;
- To identify more productive institutions and countries for potential collaboration and possible funding bodies to support the research.

To address the research gap, we synthesized publications on the application of machine learning in primary health care using synthetic knowledge synthesis (SKS) [27,29]. SKS effectively tackles the challenges posed by the rapidly increasing volume of research evidence. It employs a triangulation approach that integrates quantitative and qualitative knowledge synthesis through descriptive bibliometrics, bibliometric mapping, and content analysis, thus overcoming the limitations of traditional synthesis methods. SKS minimizes the need for manual synthesis in parts of the synthesis process and allows for the incorporation of thousands of publications, effectively addressing the sampling limitations inherent in systematic and scoping reviews, which typically synthesize only a limited number of publications. By utilizing triangulation, SKS enhances the validity, credibility, dependability, confirmability, and transferability (ecological validity) of research synthesis. Additionally, this study seeks to identify the most productive countries, institutions, funding sponsors, and prolific source titles.

## 2. Materials and Methods

SKS is a triangulation of bibliometrics, bibliometric mapping, and content analysis, which enables semi-automatic qualitative and quantitative analysis of large corpora of research publications. In this study, the SKS framework was executed by the steps presented below:

1. We harvested the research publications corpus from the Scopus bibliographic database (Elsevier, Amsterdam, the Netherlands) using the advanced search command TITLE-ABS-KEY(({machine learning} or {decision tree*} or {random forest*} or {deep learning} or {Naive Bayes} or {Neural network*} or SVM or KNN or {rough set*} or {genetic algorithm*} or {evolutionary program*}) *AND (“primary care” or “primary health”)).* The search was performed on the 15 April 2025. The Scopus bibliographic database was selected as it is the largest multidisciplinary database of peer-reviewed literature, delivering a comprehensive overview of the world’s research output in various fields, including the PubMed database. Scopus also offers simultaneous retrieval of a large amount of publications metadata and a range of useful analytical functions.
2. Descriptive bibliometrics has been performed using Scopus’s built-in functions like country and institution productivity analysis, literature production trend analysis, journal analytics, funding bodies analytics, and document type analytics.
3. The authors’ keyword landscape was generated from the entire corpus collected in Step 1 using bibliometric mapping with VOSViewer software version 1.6.20 (Leiden University, the Netherlands). VOSViewer employs text mining to recognize various text terms, specifically authors’ keywords from the keyword lists. It then uses a mapping technique called Visualization of Similarities (VoS) [30], based on the co-word analysis, to generate different bibliometric maps, in this case, the authors’ keywords landscape. Authors’ keywords were selected as meaningful units of information, referred to as codes, as they most concisely present what the authors intended to communicate to the scientific community. The number of keywords to be included in the landscape was determined by the Zipf law [31].
4. Inductive content analysis was initially conducted by examining the frequency of codes. Subsequent qualitative network analysis focused on the links and proximity between popular codes to identify distinct subnetworks representing research categories. Categories that share a common cluster were condensed together to form a cohesive research theme.

The temporal aspects of the knowledge development were analyzed by comparing the research landscapes between 2022–2023 and 2024–2025 [32].

## 3. Results and Discussion

### 3.1. Descriptive Bibliometrics

**Figure 1:**
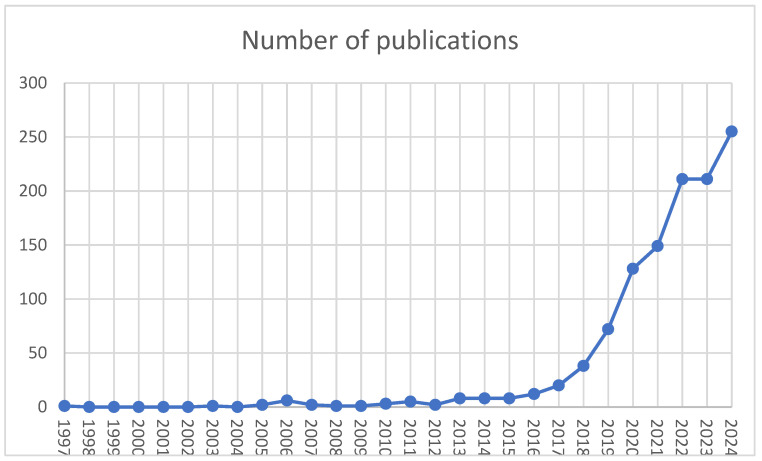
The productivity trend in the number of publications per year.

The search resulted in 1152 publications; among them were 838 journal articles, 165 conference papers, 67 reviews, 21 book chapters, 16 conference reports, 14 short papers, 10 editorials, and 8 other publications. Two papers were retracted. The first paper indexed in Scopus was a journal article on modelling obesity using the abductive network, published in 1997 [33]. Two papers on the use of rough sets, neural networks, and logistic regression to predict compliance in patients with coronary diseases [34] and a multiagent system for nurse and patient scheduling in primary care [35] were published in 2003 and 2005, respectively. After this (Figure 1), publications were sparse, not exceeding six publications per year until 2013, when the linear growth trend began, followed by exponential growth starting in 2017 and a one-year plateau in 2022. The peak productivity was reached in 2024 with 255 publications.

The most productive countries, according to the number of publications, are the United States (*n* = 392), United Kingdom (209), India (*n* = 107), China (*n* = 106), Canada (*n* = 94), Australia (*n* = 58), Spain (*n* = 45), Germany (*n* = 52), the Netherlands (*n* = 46), and South Korea (*n* = 35). The top 10 countries all have strong economies; half of them (South Korea, Australia, Canada, the Netherlands, and Germany) are among the top 10 countries in terms of the Health Care Index [36]. All of them are ranked among the top 15 most productive countries, according to the Scimago Country Rank (Elsevier, the Netherlands), in general and medical sciences. The most productive institutions are Harvard Medical School (*n* = 40), University of Toronto (*n* = 30), University of Oxford (*n* = 29), University College London (*n* = 27), Imperial College London (*n* = 26), and University of California, San Francisco (*n* = 25). All the top institutions are located in the two most productive countries, namely the United States and the United Kingdom, and are among the world’s most prolific and recognized research institutions.

The most prolific journals are *Plos One* (*n* = 33), *BMJ Open* (*n* = 26), *Scientific Reports* (*n* = 22), *Jmir Medical Informatics* (*n* = 19), and *Journal of Medical Internet Research* (*n* = 15). They are prominent and recognized international journals ranked in the first quarter in their respective categories by various impact factors. Consequently, those journals present a suitable venue for researchers to find the most relevant research and publish their own research.

#### Funding

The most productive funding sponsors are the National Institutes of Health, USA (*n* = 113); the US Department of Health and Human Services (*n* = 102); UK Research and Innovation (*n* = 54); the European Commission (*n* = 47); the National Institute for Health and Care Research, UK (*n* = 46); the National Natural Science Foundation of China (36); the Ministry of Science and Technology of the People Republic of China (*n* = 30); the Medical Research Council (*n* = 25); and the National Institute for Aging (*n* = 23). The rate of funded papers is 42%, which is relatively high compared to other research areas [37]. Information about the most prolific funding agencies is important because it enables research institutions to compete for grants, which could enable them to hire eminent researchers, provide access to advanced technology and research equipment, cooperate in major international scientific networks, gather new knowledge at top conferences, and/or hire leading external organizations to support the preparation of competitive project proposals.

### 3.2. Inductive Synthetic Knowledge Synthesis

**Figure 2:**
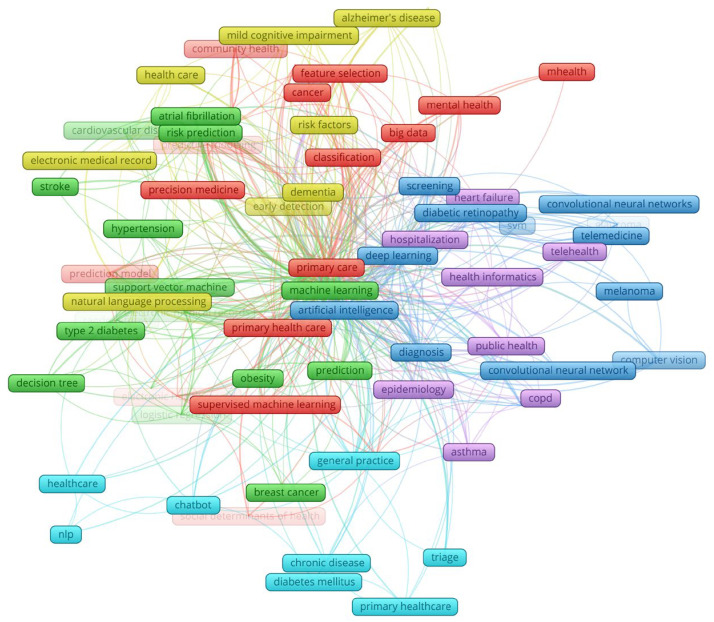
The authors’ keyword landscape on the use of machine learning in primary health care.

The publications from the corpus were analyzed using VOSviewer software (Steps 3 and 4 of the SKS framework). Text mining identified 1861 author keywords, and according to Zipf’s law, 83 were selected for the bibliometric mapping analysis. The resulting author keyword landscape is shown in Figure 2. Altogether, fourteen categories and six research themes were identified, as shown in Table 1.

#### Literature Review Based on Generated Themes and Categories

A more detailed description of themes, as seen in Table 1, based on the most influential articles from each theme, is presented below.

- **Natural language processing and clinical decision support systems in dementia, Alzheimer’s disease, and mild cognitive impairment:** Maclagam et al. [38,39] used natural language processing of free texts in electronic health records and clinical notes to identify patients with risk of dementia, Alzheimer’s, or cognitive impairment [40] in a preventive manner to shorten the length of hospitalization, delay admission to long-term care, and reduce the number of underrecognized patients with the above diseases. Artificial intelligence and speech and language processing have been used to predict the occurrence of Alzheimer’s disease [41] or cognitive decline in the context of aging to facilitate restorative and preventive treatments [42,43,44,45,46,47].
- **Optimizing health care and managing risk and patient safety in primary health with machine learning:** The use of machine learning in primary health care has recently gained popularity and promise [26,48,49,50]. Pikoula et al. [51] and Jennings et al. [52] used clustering, correspondence analysis, and decision trees on medical record data from 30961 smokers diagnosed with COPD to classify them into groups with differing risk factors, comorbidities, and prognoses. In general, AI is often used in managing COPD [53]. Oude et al. [54] developed a clinical decision support system based on various decision tree algorithms for self-referral of patients with low back pain to prevent their transition into chronic back pain. In general, AI is frequently used to support services for patients with musculoskeletal diseases [55]. Sekelj et al. [56] and performed a study to evaluate the ability of machine learning algorithms to identify patients at high risk of atrial fibrillation in primary care. They found that the algorithm performed in a way that, if implemented in practice, could be an effective tool for narrowing the population who would benefit from atrial fibrillation screening. Similarly, Norman et al. [57] used machine learning to predict new cases of hypertension. Liu et al. found that machine learning-assisted nonmydriatic point-of-care screening administered during primary care visits would increase the adherence to recommendations for follow-up eye care in patients with diabetes. On an epidemiological level, new diabetes patients were identified using stochastic gradient boosting [58]. Priya and Thilagamani [59] developed a machine learning-based prediction model to predict arterial stiffness risk in diabetes patients. Machine learning has also been used for the prediction/classification of infectious diseases [6,60], anxiety [61], COVID-19 severity [62], cancer [24,63], or even patient no-shows [13,64]. On the other hand, Evans et al. [65], Fong [66], and Govender [67] developed an automated classification of patient safety reports system using machine learning.
- **Using supervised learning and data/text mining to analyze primary health-based social determinants:** Natural language processing and big data analytics can potentially transform primary health care [68,69]. Bejan et al. [70] developed a methodology based on text mining to identify rare and severe social determinants of health in homelessness and adverse childhood experiences found in electronic health care records. Chilman et al. [71] successfully developed and evaluated a natural language processing and text mining application to analyze psychiatric clinical notes of 341,720 de-identified clinical records of a large secondary mental healthcare provider in South London to identify patients’ occupations, and Hatef et al. [72] used a similar approach on electronic health records to identify patients with high-risk housing issues. On the other hand, Scaccia [73] applied NLP to explore the concept of equity in community psychology after the COVID-19 crisis by analyzing relevant research, and Hadley et al. [74] examined the trends in health equity using text mining revenue service tax documentation submitted by nonprofit hospitals. Ford et al. [75] developed a supervised machine learning application for automated detection of patients with dementia without formal diagnosis, using routinely collected electronic health records to improve service planning and delivery of quality care. Kasthurirathne et al. [76] used random forest machine learning and NLP algorithms on integrated patient clinical data and community-level data representing patients’ social determinants of health obtained from multiple sources to build models to predict the need for referral to mental health professionals, dietitians, social workers, or other SDH services. Big data analysis using traditional non-text clinical data was used to recognize patterns of collaboration between physicians, nurses, and dietitians in the treatment of patients with type 2 diabetes mellitus; compare these patterns with the clinical evolution of the patients within the context of primary care; determine patterns that lead to the improved treatment of patients [77]; classify skin diseases [78]; predict the influx of patients to primary health centers [79]; and predict high-risk pregnancies early [80]. Garies et al. [81] used machine learning to derive health-related social determinants of primary care patients. On a larger scale, AI was used to derive social determinants of health data from medical records in Canada [82].
- **Deep learning in screening and diagnosing:** Nemesure et al. [61] developed a machine learning pipeline of machine learning algorithms, including deep learning, to predict generalized anxiety disorder and major depressive disorder using data from an observational study of 4184 undergraduate students. Deep learning for automatic image analysis [83] has been used in various studies for the early diagnosis of diabetic retinopathy in diabetic patients [84,85,86] and predicting HER2 in bladder cancer patients [87]. Convolutional neural networks were used for the early diagnosis of multiple cardiovascular diseases [88], chronic respiratory diseases [89], or melanoma [90], reaching a high accuracy between 94% and 98%. A graph convolutional network was employed for automatic diagnosis and integrated into more than 100 hospital information systems in China to improve clinical decision-making [91]. Zhang et al. [92] developed a deep learning model for sarcopenia diagnosis using clinical characteristics and laboratory indicators of aging cohorts.
- **Health informatics in primary health:** The COVID-19 pandemic additionally triggered the employment of machine learning in primary health for various applications, such as the management of COVID-19 with intelligent digital health systems [93], chatbots to classify patient symptoms and recommendations of appropriate medical experts [94], the evaluation of vaccine allergy documentation [95], predicting the need for hospitalization or home monitoring of confirmed and unconfirmed coronavirus patients [96], and predicting the severity of COVID-19 among older adults [97]. From the epidemiological viewpoint, machine learning in primary health has been used for frailty identification [98], heart failure prediction [99], determining the incidence of infectious diseases from routinely collected ambulatory records [100], and identifying psychological antecedents and predictors of vaccine hesitancy [101]. On the other hand, machine learning has been used for clinical decision support for childhood asthma management [102] and predictive analytics in nursing [103]. In general, health informatics supported by machine learning can significantly improve primary health care [104,105].
- **Chatbots in primary health care:** In the last four years, chatbots have become more frequently used in primary health care [106,107,108]. They are used to make health care systems more interactive by using NLP to understand patients’ queries and give suitable responses [109,110,111] or even to virtualize primary health care [112], such as detecting possible COVID-19 cases and guiding patients [113]. Further examples include using chatbots to try to persuade smokers to quit smoking [114]; help patients with anxiety, depressive symptoms, or burnout syndrome [115,116]; provide support to patients with chronic diseases [117]; detect early onset of cognitive impairment [118] and suicidal intentions [119]; guide mothers or family members about breastfeeding [120]; or address patient inquiries in hospital environments [121].

### 3.3. Deductive Synthetic Knowledge Synthesis

The deductive part of the SKS analysis revealed that the benefits of using machine learning in primary health care emerge at three beneficiary levels: the patient level, the health care provider level, and the health care system level. Potential patient benefits include improved quality of life, patient-centered care and patient safety, early diagnosis, identification of high-risk patients, screening effectiveness, and more effective and efficient prevention and treatment of diseases. The most targeted diseases mentioned in over five publications were COVID-19, dementia, cardiovascular diseases, depression, diabetes, Alzheimer’s, asthma, suicide, mental health, mild cognitive impairment, and cancer. Potential benefits for health care providers include facilitated referrals, enhanced quality of primary health delivery, better communications, and reduced workload. Potential benefits for health care systems include enhanced population-based screening, surveillance, predictions, more effective and efficient decision-making on the system level, better management of health institutions, and reduced economic burden.

The most frequently used machine learning approaches were deep learning, decision trees, logistic regression, convolutional neural networks, neural networks, and random forests.

A comparison of the 2022–2023 and 2024–2025 research landscapes revealed that the content of the research on machine learning in primary health care has not changed much in recent years. However, the focus shifted from COVID-19, Alzheimer’s, digital health transformation, classification and decision support, big data, eHealth, and convolutional networks to natural language processing, chatbots, cardiology, general practice, quality improvement on an individual level, and computer vision. The shift in focus might have occurred due to the advancement in natural language processing. Earlier AI focused more on structured data (like medical images or big data analytics); however, the advent of large language models enabled the potential for AI to understand and generate human language. This makes chatbots and conversational AI much more viable for patient interaction, administrative tasks, and providing basic health information. COVID-19 and Alzheimer’s were significant research priorities due to their global impact and the urgent need for solutions. AI’s early promise in analyzing vast datasets for drug discovery, vaccine development, or disease prediction made it a natural fit. However, unique challenges, including high patient volumes, diverse conditions, and the need for efficient triage and decision support, triggered the development of AI applications directly applicable to common primary care scenarios and the integration of AI into the daily workflow of primary care providers. An additional reason for the shift might be the emergence of user-centric designs and the growing understanding that AI tools need to be practical and user-friendly for health care professionals. In essence, the shift signifies a move from exploring the broad potential of AI in health care to focusing on more targeted, mature, and practically applicable solutions that address the specific needs and challenges of primary health care.

SKS also identified some challenges for the successful and widespread use of machine learning in primary health care, such as how to more actively involve end users; how to make a paradigm shift from technology-centered to human-centered design approaches; how to ensure cost-effectiveness and performance of machine learning-based primary health care systems; how to overcome ethical, standardization, and legal aspects (i.e., data protection and security); how to increase the AI health literacy of patients; and finally, how to validate the quality and validity of the input data for machine learning algorithm training. Among these challenges, the ethical and regulatory barriers might be the hardest to overcome. If the training datasets are not representative of diverse populations or are, for example, prioritizing cost-saving measures, machine learning can perpetuate and even amplify existing societal biases and health disparities. Another concern might be that machine learning can inadvertently “memorize” sensitive health information, which can lead to erosion of trust in patient–provider relationships. Finally, machine learning might diminish the human elements of health care, reducing empathy in patient–health care professional interactions.

### 3.4. Strengths and Limitations

The study’s main strength is that it is the first holistic and systemic analysis of the content of research publications dealing with the use of machine learning in primary health. Another strength is that thematic analysis was performed using a novel synthetic knowledge synthesis approach. One possible limitation is that the analysis was limited to publications indexed in Scopus only; however, because Scopus covers the most extensive and complete set of information titles, we believe we analyzed most of the critical peer-reviewed publications. Nevertheless, the results might be slightly different if other bibliographic databases were also considered.

Our study presents the first comprehensive and holistic study combining both quantitative and qualitative analyses of the use of machine learning in primary health. This topic was also chosen because machine learning will likely be an essential and crucial approach to developing better primary health systems and services. This study might help primary health professionals gain new insights into this topic, deepen their knowledge, or inform them about the trends and essential themes regarding the use of machine learning in primary health care.

## 4. Conclusions

Our SKS study presented the extent and variety of machine learning use in primary health care. We showed that the use of machine learning and the underlying research efforts are growing exponentially, while also revealing several challenges. We summarize that the use of machine learning in primary health care aims to address the significant global burden of so-called “missed diagnostic opportunities”, which mainly occur due to inevitable human limitations, and enhance diagnostic, screening, treatment, and management decision-making to improve primary health care, while minimizing possible adverse effects on patients.

## Figures and Tables

**Table 1:** Representative keywords, categories, and themes of use of machine learning in primary health care research publications.

Cluster Color	Representative Author Keywords (The Number in Parentheses Represents the Number of Occurrences in Publications)	Categories	Theme
Yellow (12 author keywords)	Natural language processing (28); Dementia (13); Risk factors (9); Mild cognitive impairment (9)	Natural language processing of medical records for clinical decision support in dementia health care; Identification of risk factors for early detection of dementia, Alzheimer’s, and mild cognitive impairment with natural language processing	Natural language processing and clinical decision support systems in dementia, Alzheimer’s disease, and mild cognitive impairment
Green (19 author keywords)	Machine learning (239); Electronic health records (47); Prediction (19); Risk prediction (13); Atrial fibrillation (13)	Use machine learning algorithms like support vector machines, random trees, decision trees, and logistic regression on electronic health records in cardiovascular diseases, diabetes, and other chronic diseases; Machine learning in risk prediction and prediction in general; Improve patient safety with machine learning	Optimizing health care and managing risk and patient safety in primary health with machine learning
Red (20 author keywords)	Primary care (89); Primary health care (24); Depression (16); Classification (15); Supervised machine learning (8); Precision medicine (7); Mental health (7); Big data (7)	Using text mining and classification in primary, community, population, and mental health to improve social determinants; Supervised machine learning in primary health care delivery; Big data and data mining in primary care; Precision medicine and depression	Use of supervised learning and data/text mining to analyze primary health-based social determinants
Blue (13 author keywords)	Artificial intelligence (99); Deep learning (77); Diagnosing (29); Screening (23); Convolutional neural networks (18); Diabetic retinopathy (15); Telemedicine (8)	Artificial intelligence and deep learning in screening and diagnosing; Deep learning with convolutional networks in computer vision; Screening of diabetic retinopathy and glaucoma with deep learning; Use of artificial intelligence in telemedicine	Deep learning in screening and diagnosing
Violet (10 author keywords)	COVID-19 (24); Public health (14), Telehealth (8); Epidemiology (8); Health informatics (7);	COVID-19 and telehealth; Use of health informatics in epidemiology; Health informatics and asthma	Health informatics in primary health
Light blue (9 author keywords)	General practice (12); Suicide (8); Chatbot (5); NLP (5)	Chatbots in general practice in primary health; Chatbots and NLP	Chatbots in primary health care

## Data Availability

No new data were created or analyzed in this study. Data sharing is not applicable to this article.
